# Effect of Aging on NK Cell Population and Their Proliferation at Ex Vivo Culture Condition

**DOI:** 10.1155/2018/7871814

**Published:** 2018-08-02

**Authors:** Sellamuthu Subbanna Gounder, Basri Johan Jeet Abdullah, Nur Ezzati Izyan Binti Mohd Radzuanb, Farah Dalila Binti Mohd Zain, Nurhidayah Bt Mohamad Sait, Corine Chua, Baskar Subramani

**Affiliations:** ^1^Nichi-Asia Life Science Sdn. Bhd., No. 57, Block F, Jalan Teknologi 3/9, Bistari De Kota, Kota Damansara, PJU 5, 47810 Petaling Jaya, Selangor, Malaysia; ^2^Department of Biomedical Imaging, University of Malaya, 50603 Kuala Lumpur, Malaysia

## Abstract

Age-associated changes in natural killer (NK) cell population, phenotype, and functions are directly attributed to the risk of several diseases and infections. It is predicted to be the major cause of the increase in mortality. Based on the surface density of CD56, NK cells are subdivided into two types, such as CD56^bright^ and CD56^dim^ cells, which represent cytokine production and cytotoxicity. In our study, we have examined the age-associated changes in the NK cell population and their subsets at different age groups of males and females (at a range from 41 to 80 years). We found that the total lymphocyte count significantly dropped upon aging in both genders. Although, the level of total immune cells also dropped on aging, and surprisingly the total NK cell population was remarkably increased with the majority of NK cells being CD56^dim^. Subsequently, we evaluated the proliferation potential of NK cells and our results showed that the NK cell proliferation ability declines with age. Overall, our findings prove that there is an increase in the circulating NK cell population upon aging. However, the proliferation rate upon aging declines when compared to the young age group (<41 yrs).

## 1. Introduction

Natural killer (NK) cells are considered the primary defense lymphocyte against virally infected and virally transformed cells. The coverage of their defense system has been extended to include antimicrobial response [1, 2], elimination of senescent cells [3], resolution of inflammation [4, 5], and induction of adoptive immune response [6, 7]. These potential NK cells are identified as CD56 positive and CD3 negative, and they are located in the majority of our organs and tissues, especially peripheral blood, skin, lymph nodes, bone marrow, thymus, liver, intestines, lungs, uterus, and so on. NK cells are classified into two distinct populations based on the surface density of their CD56 expression, namely, CD56^bright^ and CD56^dim^ NK cells; both of them have unique functional characteristics [8]. Briefly, CD56^bright^ NK cells represent a minimal (10%) population in the circulatory system and have low or no cytotoxic response. However, this NK cell subset produces an array of cytokines and chemokines which influence immunomodulation and thus these cells are commonly referred to as “cytokine producers.” In contrast, CD56^dim^ NK cells are predominant (90%) in the circulatory system and are potent mediators of natural and antibody-dependent cytotoxicity [9, 10]. The absolute number of NK cells and their NK^bright^ : NK^dim^ ratio is impaired upon aging which could be a reason why elderly people become more prone to several diseases, infections, and cancers.

Physiological aging is an evolutionarily conserved process which is associated with a defective or impaired function of immune cells, including NK cells. The impaired function of NK cells is known as NK cell immunosenescence. Age-associated NK cell immunosenescence contributes to the higher incidence of viral infection and cancer induction. Also, NK cell-mediated elimination of senescent cells declines on aging and results in the accumulation of aged cells in tissue or organs which impairs tissue homeostasis and their function. NK cell-mediated elimination of senescent cells is a direct elimination (migration, recognition, binding, and elimination of their targets) process which is accomplished by the NK^dim^ cell through the granule exocytosis pathway [3]. However, a recent finding speculated that upon aging the expression pattern of perforin and migration ability declines in NK^dim^ cells, which directly influences the NK cell-mediated cytolysis on the senescent cell. Unlike the NK^dim^ cell, the total population, phenotype, and functions of NK^bright^ cells decline due to aging, which is attributed to poor immunomodulation, poor resolution of inflammation, and poor induction of adaptive immunity. One study examined the effect of aging on the cytokine production of NK^bright^ cells and reported that the production of cytokines (IFN-*γ*, MIP-1*α*, IL-8, etc.) is significantly lower in older NK^bright^ cells than in younger NK^bright^ cells [11].

Several studies signify that the NK cell number and subpopulations vary upon aging with an increase of the CD56^dim^ population and a decrease of the CD56^bright^ population [12–15]. In elderly subjects, decreased NK cell activity has been shown to be associated with an increased incidence and severity of many diseases such as coronary heart disease [16], liver fibrosis [17], infectious diseases, and cancer [18]. Thus, active NK cells with higher levels of activity are a prerequisite of natural well being [19, 20]. In cancer patients, we have proven that the higher number of NK cell leads to better quality of life [21]. NK cells are important in the innate immune system to host the early defense. They also have a unique function as the primary source of immunoregulatory cytokines and they partially regulate monokine production. In order to do that, in the present study, we have directly compared the absolute number of NK cells and their subsets at different age points in both male and female populations and also examined the NK cell expansion potential of the respective age groups with a well-defined protocol.

## 2. Materials and Methods

### 2.1. PBMC Isolation and Quantification

Peripheral blood was obtained from 40 donors from different age groups (ranging from 41 to 80 years old), with no significant medical illnesses or a history of cancer. All enrolled donors signed informed consent forms approved by the medical research ethics committee, University Malaysia Medical Centre, Malaysia. NK cell-containing peripheral blood mononuclear cells (PBMCs) were isolated by lymphoprep (Axis-Shield Diagnostics Ltd., Oslo, Norway) density gradient medium. Isolated PBMCs were washed twice with magnesium and calcium free phosphate buffered saline (PBS). The PBMCs were counted in an automated cell counter (Nexcelom Bioscience, USA).

### 2.2. NK Cell *In Vitro* Expansion

The isolated PBMCs were finally resuspended in NK cell expansion media containing 10% autologous plasma and 700 IU/ml of IL-2. Suspended PBMCs were seeded at a density of 1 × 10^6^/ml as per the protocol described by us earlier [22].

### 2.3. Flow Cytometry Analysis

Flow cytometry analysis was performed to qualify and quantify the NK cell population in peripheral blood and cultured cells using NK specific antibodies especially CD56-PE and CD3-PC5 (Beckman Coulter Inc., USA). Staining was performed as per the manufacturer's instruction. Stained cells were washed and resuspended in PBS and analyzed by flow cytometry (FC 500, Beckman Coulter Inc., USA). The acquired data were analyzed using CXP software provided by the manufacturer.

### 2.4. Statistical Analysis

Data from each group were expressed a mean and standard error (SE) of at least three separate experiments performed. Statistical comparison between groups was analyzed using Student's *t*-test. A value of ^#^^,^^@^^,^^∗^*P* < 0.05 was considered to be statistically significant.

## 3. Results

### 3.1. Effect of Aging on Lymphocyte Count

**Figure 1:**
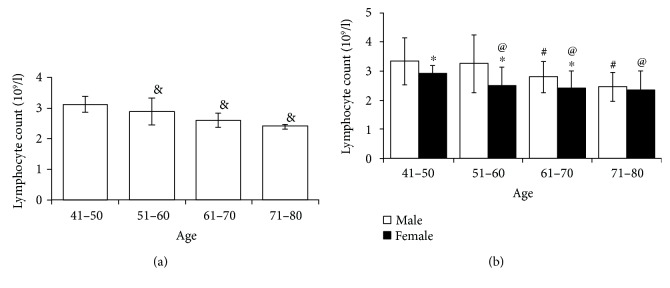
Total lymphocyte count was performed by an autoanalyzer. (a) Upon aging, the lymphocyte count declined remarkably in both genders. (b) However, the lymphocyte count was considerably lower in the female gender at almost all different age points compared with that of the male gender. *P* > 0.05. ^&^Between age groups. ^∗^Within the same age group. ^#^Among males compared to ages 41–50. ^@^Among females compared to ages 41–50.

The study population consists of 40 individuals (*n* = 20 male and *n* = 20 female) who were segregated into four groups with each group containing 5 males and 5 females; Group 1 (41–50 years old), Group 2 (51–60 years old), Group 3 (61–70 years old), and Group 4 (71–80 years old). The absolute lymphocyte count was performed at different age groups using an autoanalyzer and the results showed that the average lymphocyte count significantly decreased upon aging (Figure 1(a)). The average quantity of lymphocytes per liter of PB was 3.12×10^9^ ± 0.25, 2.8×10^9^ ± 0.13, 2.6×10^9^ ± 0.08, and 2.4×10^9^ ± 0.09 at the ages of 41–50, 51–60, 61–70, and 71–80 years old, respectively. When compared to males, lymphocyte count was considerably lower in females in all age groups (Figure 1(b)). These results suggest that females are highly susceptible to diseases and accompany faster aging than males.

### 3.2. Effect of Aging on NK Cell Population

**Figure 2:**
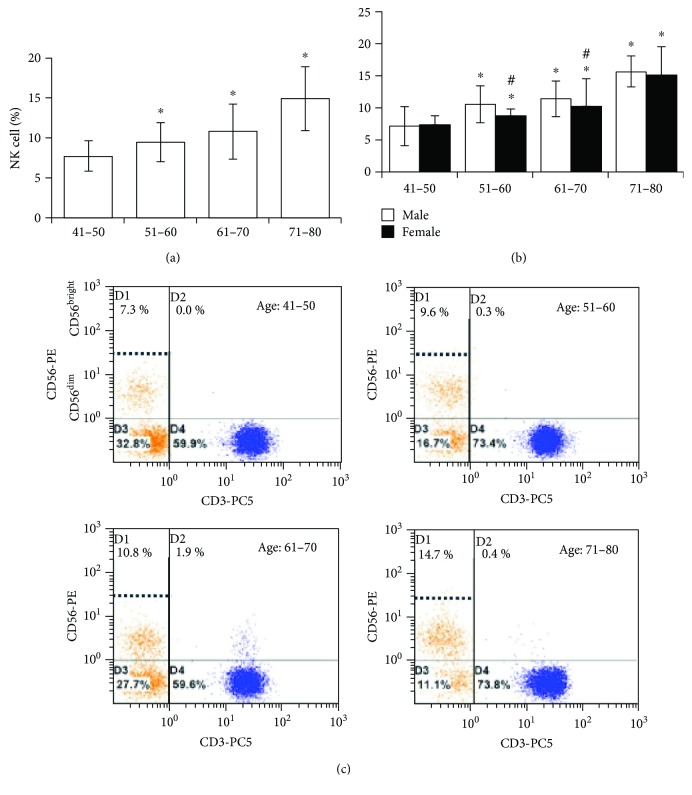
NK cell population was quantified by flow cytometry. (a) The NK cell population was sequentially increased on aging in both genders. (b) However, the NK cell population was considerably decreased in the female gender at almost all different age points compared with that in the male gender. (c) Flow cytometry images of CD56-positive NK cell populations at different age points. *P* > 0.05. ^∗^Between age groups, compared to the corresponding 41–50 age group. ^#^Among males compared to ages 41–50.

The percentage of NK cells was analyzed in the peripheral blood of each age group. The median percentage of CD56^+^NK cells was significantly increased upon aging. The expression patterns of CD56 were 7.3 ± 1.5% (41–50 years old), 9.6 ± 1.8% (51–60 years old), 10.8 ± 2.3% (61–70 years old), and 14.7 ± 3.0% (71–80 years old) (Figure 2(a)). After the age of 50, the CD56^+^ NK cell population was significantly decreased in the female gender than in the corresponding male gender (Figure 2(b)). Next, we analyzed the NK cell subsets of CD56^+^NK^dim^ and CD56^+^NK^bright^ in different age groups. There was a significant variation observed in the expression pattern of CD56^+^NK^dim^ and CD56^+^NK^bright^ in all age groups. However, the matured CD56^+^NK^dim^ cell population was predominantly present in all age groups, while, very minimal or a negligible amount of the immature CD56^+^NK^bright^ cell population was found in all age groups (Figure 2(c)). This information demonstrates that an increased population of mature NK cells (CD56^+^NK^bright^) among aging populations may be a result either of an accumulation of long-lived NK cells or an impaired routine process of elimination of increased-senescence cells.

### 3.3. Effect of Aging on NK Cell Expansion

**Figure 3:**
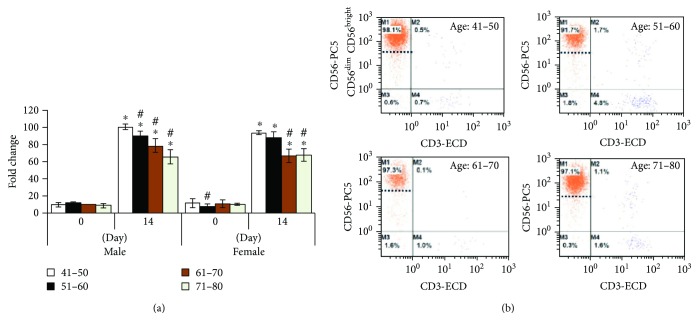
NK cell growth and their subsets were analyzed by flow cytometry. (a) NK cells from all age groups were dramatically divided and manufactured to almost more than 90% purity. Cell fold was significantly increased at all age groups in both genders after 14 days of culture. In comparison with aging, NK cell growth in elderly people was significantly lower than that in younger people. (b) Flow cytometry analysis of 14-day cultured CD56^bright^ and CD56^dim^ NK cell subsets at different age settings. *P* > 0.05. ^∗^Between before and after cultured NK cells. ^#^Between age groups.

An average of more or less a similar number of PBMCs from different age groups were seeded on day 0 and then cells were allowed to grow for 14 days with NK cell-specific culture medium. After 14 days, the count was performed using the trypan blue exclusion method. NK cells were grown successfully in all age groups with more than 90% purity. With regard to NK cell fold change between different age groups, after 14 days of culture, the NK cell fold changes were significantly decreased upon aging in both male and female genders (Figure 3(a)). Flow cytometry analysis revealed that most of the replicated NK cells are CD56^+^NK^bright^ cells at all age groups, while a very minimal number of CD56^+^NK^dim^ cells were observed in all age groups (Figure 3(b)). These results suggest that CD56^+^NK^bright^ cells are equally expressed in all age groups even though NK cell fold changes between different age groups significantly decreases upon aging in both males and females.

## 4. Discussion

Manipulations are more predominant in the case of aging with newer approaches and strategies. The most evident modification is the deregulation of the immune function which contributes to the increased vulnerability to infection of the aged people [19]. The study has shown that the low number of NK cells is associated to a high risk of mortality rate in elderly individuals compared with individuals who have a higher number of NK cells [23]. In addition, a significant decrease in cytotoxicity was noted in the cells isolated from elderly donors [24]. On the other hand, studies have reported that NK cells produced better cytotoxicity in healthy aging individuals [25, 26]. Our data shows that total peripheral blood lymphocyte count declined with age. Chidrawar et al. also proved the decline of lymphocytes upon aging [27]. Conversely, the total lymphocyte count was higher in males compared to females. The intake of alcohol, smoking, and stress levels were associated with higher lymphocyte counts in males [28].

The results reveal that the number of CD56^bright^ NK cells declines with age, which may have a huge proposition for NK cell function in the elderly individuals [27]. Our data is also consistent with an earlier study that showed that the levels of CD56^bright^ NK cells were lower in all four groups compared with CD56^dim^. The total NK cell population was higher with advancing age; however, the age-related increase may be an accumulation of long-standing NK cells in older adults [29]. The lytic capacity was lower, although a high number of NK cells were present in an elderly cohort [24].

In this study, we used the optimized protocol to produce large numbers of activated NK cells from healthy donors of the different age cohorts. The use of clinical gate IL-2 plays essential roles in NK cell development, activation, and expansion [30, 31]. In the presence of IL-2, we obtained optimal fold expansion from 60- to 100-fold and expanded NK cells were mainly composed of a CD56^bright^ population which is responsible for innate immune response and cytokine production [27, 32]. However, the population of elderly cohort cells did not reach 100-fold within 14 days. The low number of immature CD56^bright^ cells could delay the fold changes in an elderly cohort [24].

In our study, we have shown that the number of lymphocytes and CD56^bright^ NK cells decreased with age while the number of CD56^dim^ NK cells increased. However, after *in vitro* activation with IL-2, the expressions of CD56^bright^ cells were higher and the fold changes increased. Overall, our optimized protocol managed to expand the number of CD56^bright^ NK cells. However, a more elaborate approach needs to be carried out to evaluate the cytotoxic activity of these variant NK cell populations.

## Data Availability

The datasets generated and/or analyzed during the current study are not publicly available due to confidentiality, but are available from the corresponding author on reasonable request.
